# Drug**-**drug interactions between direct oral anticoagulants and anticonvulsants and clinical outcomes: A systematic review

**DOI:** 10.1016/j.rpth.2023.100137

**Published:** 2023-03-28

**Authors:** Matteo Candeloro, Stephanie Carlin, Michelle J. Shapiro, James D. Douketis

**Affiliations:** 1 Department of Innovative Technologies in Medicine and Dentistry, “G. D'Annunzio” University, Chieti, Italy; 2 Department of Medicine, McMaster University, Hamilton, Ontario, Canada; 3 Department of Pharmacy, Hamilton Health Sciences, Hamilton, Ontario, Canada; 4 Department of Medicine, Division of Neurology, McMaster University, Hamilton, Ontario, Canada

**Keywords:** anticoagulants, anticonvulsants, atrial fibrillation, drug interactions, thrombosis

## Abstract

**Background:**

Direct oral anticoagulants (DOACs) are widely used in patients with atrial fibrillation and venous thromboembolism. However, DOACs have important potential drug-drug interactions (DDIs) with several classes of drugs. In particular, antiepileptic (AE) drugs may induce cytochrome P450 3A4 or P-glycoprotein. Co-administration of DOACs and AE drugs may result in lower DOAC drug levels and reduced DOAC efficacy. However, the clinical significance of such DDIs is uncertain.

**Objectives:**

The aim of this systematic review was to generate an updated review of these DDIs and their clinical relevance, given the rapidly evolving knowledge relating to DOAC and AE DDIs.

**Methods:**

We searched the MEDLINE and Embase databases for studies reporting clinical adverse outcomes (thrombotic events, bleeding events, and all-cause mortality) in patients concomitantly taking DOACs and AE drugs.

**Results:**

We retrieved 874 studies of which 15 were deemed eligible for this review, including 4 congress abstracts, 3 case reports, 2 letters to the editor, 5 retrospective cohorts, and 1 prospective cohort study. No randomized clinical trials were found. Most of the included studies reported thrombotic events, 3 studies reported major bleeding, and one study reported all-cause mortality associated with DOAC and AE drug administration. Substantial differences in the study designs did not allow for a meta-analysis to be performed.

**Conclusion:**

The current literature assessing these adverse clinical outcomes from DOAC and AE drug co-administration is limited. Although the available data point to a possible increased risk of thrombotic events, they are insufficient to draw definitive conclusions. Well-designed clinical studies are of utmost importance.

## Essentials

• Direct oral anticoagulants (DOACs) and antiepileptic drugs are often co-administered.
• Co-administration of DOACs and antiepileptic drugs may result in lower DOAC efficacy.
• These drug-drug interactions (DDIs) may result in adverse clinical events such as thrombosis.
• Current data are insufficient to draw conclusions about these DDIs and adverse clinical events.

## 1 Introduction

Over the past decade, direct oral anticoagulants (DOACs), including dabigatran, rivaroxaban, apixaban, and edoxaban, have become the recommended, first-line therapy for stroke prevention in atrial fibrillation (AF) and for the treatment and prevention of venous thromboembolism (VTE) [1,2]. Despite fewer drug-drug interactions (DDIs) with DOACs than with vitamin K antagonists, concerns remain regarding several possible clinically important DOAC DDIs. DOAC absorption, metabolism, and elimination are mediated by P-glycoprotein (P-gp) and cytochrome P-450 3A4 (CYP3A4). All DOACs are subject to P-gp efflux in the gut and kidney; however, have differing reliance on CYP3A4 for drug metabolism (dabigatran none, edoxaban less than 4%, rivaroxaban 18%, and apixaban 25%) [1,3]. The co-administration of DOACs and inducers of P-gp and CYP3A4 may therefore reduce the bioavailability or increase the clearance of DOACs, with the potential to increase the risk of stroke and thromboembolism. By contrast, the co-administration of DOACs and inhibitors of P-gp and CYP3A4 may increase DOAC bioavailability or reduce DOAC clearance and lead to DOAC bioaccumulation, with the potential to increase the risk of bleeding complications.

Antiepileptic (AE) drugs, including carbamazepine, phenytoin, phenobarbital, and primidone, are strong inducers of CYP3A4 and P-gp, and product monographs recommend avoidance of concomitant use. Valproate may be a mild inducer of CYP3A4, and levetiracetam may interact with DOACs through an unknown mechanism, but product monographs do not address these potential interactions. All of these AE drugs are commonly used medications for the treatment of seizures, although some are also used for pain (such as carbamazepine) or psychiatric disorders [1,4,5]. The AE drugs lamotrigine, gabapentin, and lacosamide are considered safer than other AE drugs because of their theoretical lack of interaction with DOACs with additional evidence from a case-control study from the article by Gronich et al. [6]; however, these assumptions are still a matter of debate. In the updated European Heart Rhythm Association guidelines on DOAC use in AF, close monitoring of patients who are receiving concomitant AEs is recommended, possibly in specialized anticoagulant management clinics. Moreover, they highlight that DOAC plasma-level measurements, although feasible, have not yet been standardized [1]. Therefore, the use of DOAC levels for therapeutic adjustment or discontinuation of therapy in patients co-treated with AE drugs requires further study.

Data from a US commercial and Medicare Advantage health insurance claims database study assessing the co-administration of DOACs and other oral anticoagulants with AE drugs highlighted a marked increase in concomitant use between 2011 and 2018 [7]. These findings highlight the need to clarify the existence and clinical significance of DDIs between DOACs and AE drugs.

Taha et al. [8] previously performed a systematic review assessing DOAC and AE DDIs, concluding that enzyme-inducing AE drugs reduced the effectiveness of DOACs, although the overall quality of the records was poor and data relating to edoxaban were lacking. The omission of edoxaban-related data is clinically important because this DOAC is increasing in use and has minimal dependence on CYP3A4 for metabolism and therefore may be a safer DOAC when combined with AE drugs that affect this metabolic pathway [1].

Close monitoring of patients who are concomitantly taking DOAC and AE drugs is recommended; however, there still is no consensus on whether co-prescription of DOACs and AE drugs in clinical practice is appropriate or should be avoided [1]. Given the rapidly evolving knowledge relating to DOAC DDIs, the aim of this study was to provide an expanded and updated systematic review and summarize possible clinical implications of DOAC and AE drug co-administration.

## 2 Methods

The MEDLINE and Embase databases were systematically searched by title, abstract, and full-text for studies focusing on DOAC and AE DDIs and related clinical adverse events (thrombosis, major bleeding event, or mortality) from March 2019 until December 2022 to build on a previous meta-analysis [8]. Case reports, case series, observational studies, and randomized clinical trials were included. The quality of the included cohort studies was evaluated using the Newcastle-Ottawa scale for observational studies [9]. Reviews, meta-analyses, and articles in a language other than English were excluded. Additionally, records with insufficient information regarding the type of DOAC and/or the type of AE administered, as well as those studies reporting only anti-Xa serum concentration were excluded. Moreover, articles that included patients affected by COVID-19 were excluded to avoid additional confounding.

The DOACs assessed were dabigatran, rivaroxaban, apixaban, and edoxaban, and the AE drugs assessed were phenobarbital, phenytoin, primidone, valproate, carbamazepine, ethosuximide, brivaracetam, clobazam, eslicarbazepine, felbamate, gabapentin, pregabalin, lacosamide, lamotrigine, levetiracetam, oxcarbazepine, perampanel, tiagabine, topiramate, vigabatrin, zonisamide, rufinamide, and cenobamate. Because there is still little clarity on the subject and previous works were based mainly on case reports, we decided to include all of the AE drugs used in clinical practice even when, at least theoretically, there should be no DDI with DOACs.

The search was independently performed by 2 authors (M.C. and S.C.), and conflicts were resolved through discussion and consensus.

## 3 Results

The literature search strategy is described in Supplementary Table 1, and the article screening process is shown in the Figure. The interrater percentage of agreement was high (95% for the title and abstract screening and 92% for the full-text screening).

**Figure:**
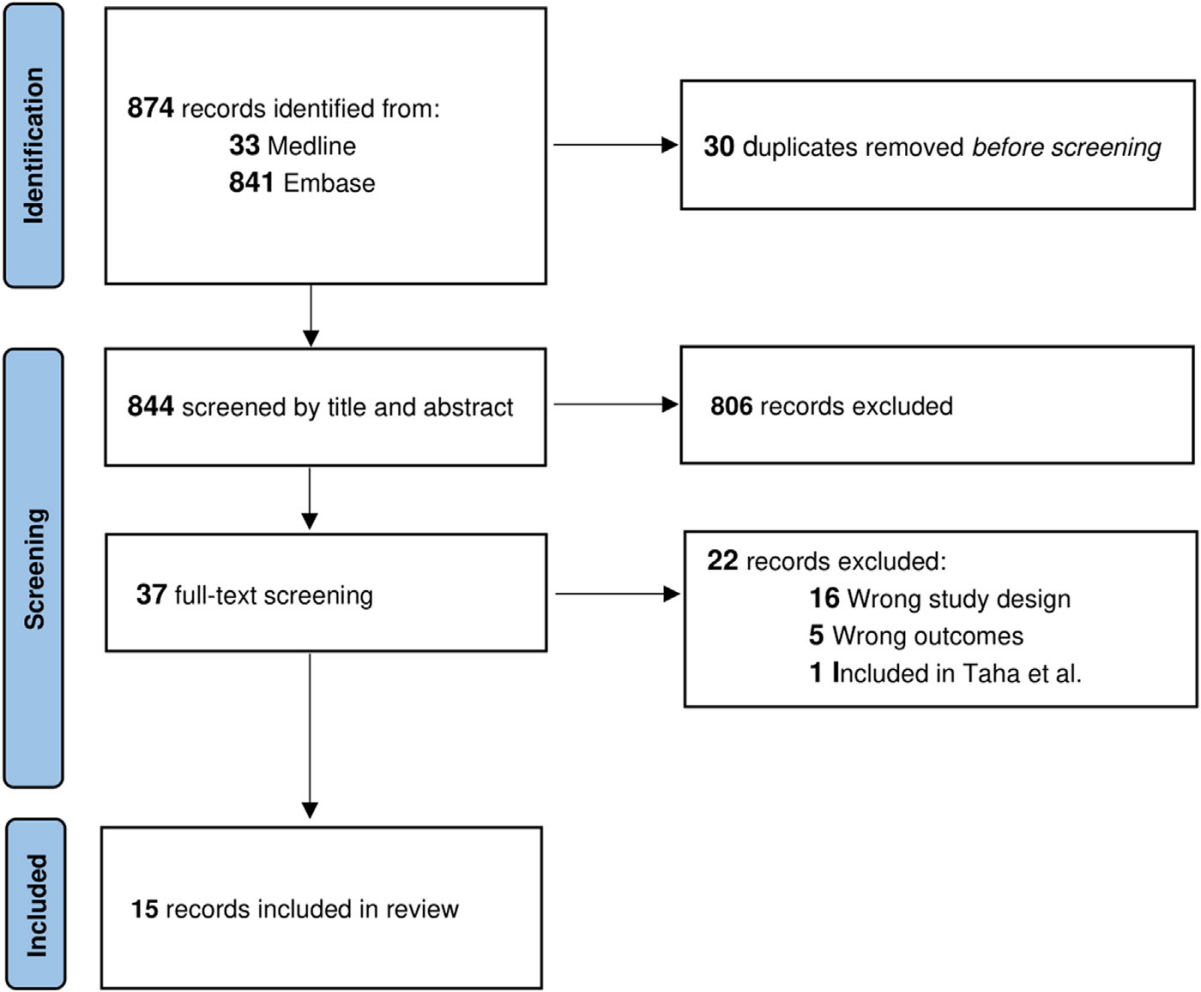
Preferred Reporting Items for Systematic Reviews and Meta-Analyses flow chart.

In total, 874 articles were retrieved from the search, and from this group, we identified 15 relevant studies, including 4 congress abstracts [[10], [11], [12], [13]], 3 case reports [[14], [15], [16]], 2 letters to the editor [17,18], 5 retrospective cohorts [6,[19], [20], [21], [22]], and one prospective cohort study [23] (Table).

**Table:** Summary of the included studies.

	Publication year	Study design	Number of patients	DOAC examined	AE examined	Outcome	Results description
Di Gennaro [16]	2019	Case report	1	Apixaban Edoxaban	Carbamazepine	Thrombotic event	An episode of TIA during apixaban and carbamazepine treatment. No further thrombotic events after 18 mo of edoxaban treatment.
Perlman [22]	2019	Retrospective cohort	9693	Rivaroxaban Apixaban	Carbamazepine Phenytoin Phenobarbital Valproic acid Lamotrigine Levetiracetam	Thrombotic event	ORs for thromboembolic or ischemic events in the Food and Drug Administration Adverse Event Reporting System (FAERS)
Barbar [10]	2020	Congress abstract	14	Dabigatran Rivaroxaban Apixaban Edoxaban	Levetiracetam Valproic acid Phenobarbital Phenytoin	Thrombotic event	During a follow-up of 10 mo (range, 1-27 mo), none of the patients co-treated with a DOAC and AE experienced cerebrovascular events and/or venous thrombosis.
Langenbruch [15]	2020	Case report	1	Rivaroxaban	Valproic acid, Lamotrigine	Thrombotic event	Multiple DVT episodes. The patient had protein S-deficiency, hyperhomocysteneimia, and elevated lipoprotein(a).
Paciullo [14]	2020	Case report	1	Rivaroxaban	Levetiracetam	Thrombotic event	Recurrent transient ischemic attacks in patients with AF and Janus Kinase 2 positive thrombocythemia.
Paskaleva [11]	2020	Congress abstract	1	Dabigatran Rivaroxaban	Valproic acid	Deep vein thrombosis	30-y-old man on treatment with valproic acid who started rivaroxaban following a DVT episode. Because of no improvement, rivaroxaban was replaced with dabigatran. Eventually, dabigatran was replaced with VKA with DVT signs and symptoms improvement.
Shah [12]	2020	Congress abstract	1	Dabigatran	Phenytoin	Cerebral vein thrombosis	25-y-old man on treatment with phenytoin for recurrent seizures who developed cerebral venous thrombosis. Treatment with dabigatran was started, but the patient experienced a worsening of the cerebral venous thrombosis.
Wang [21]	2020	Retrospective cohort	104,319	Dabigatran Rivaroxaban Apixaban	Carbamazepine Gabapentin Lamotrigine Levetiracetam Oxcarbazepine Phenobarbital Phenytoin Pregabalin Topiramate Valproic acid Zonisamide	Major bleeding	Among 104,319 patients on treatment with a DOAC, the authors compared the incidence of major bleeding between patients on treatment with a DOAC alone with those on a DOAC and one of the listed AE drugs. The concurrent use of a DOAC and valproic acid, phenytoin, or levetiracetam was associated with a higher risk of major bleeding. No data were reported for edoxaban.
Zhou [19]	2020	Retrospective cohort	45,719	Dabigatran Rivaroxaban Apixaban Edoxaban	Topiramate Phenytoin	Thrombotic event	Patients with at least one thrombotic event had RR 2.04 (95% CI, 1.02-4.05) to be on dabigatran and topiramate. Patients with at least one thrombotic event had RR 2.39 (95% CI, 1.33-3.29) to be on rivaroxaban and phenytoin. No data were reported for apixaban and edoxaban.
Giustozzi [23]	2021	Prospective cohort	91	Dabigatran Rivaroxaban Apixaban Edoxaban	Levetiracetam Valproic acid Phenobarbital Carbamazepine Oxcarbazepine Pregabalin Phenytoin Gabapentin Lacosamide Lamotrigine Topiramate	Thrombotic event Major bleeding	After a median follow-up of 17.5 mo, there was a 5.7% patient-year incidence of stroke/TIA/SE and 1.9% patient-year incidence of major bleeding.
Gronich [6]	2021	Retrospective cohort	89,284	Dabigatran Rivaroxaban Apixaban	Carbamazepine Phenytoin Phenobarbital Primidone Topiramate Lamotrigine Gabapentin	Thrombotic event	Compared with event-free matched controls, cases with new stroke/SE had an adjusted OR of 2.18 (95% CI, 1.55-3.06) of taking a PGP/CYP3A4 inducer, including hypericin herb and rifampicin. Taken singularly, carbamazepine and phenytoin held their association, whereas phenobarbital, primidone, and topiramate did not. Valproic acid and levetiracetam were both associated with stroke/SE events. Lamotrigine, and gabapentin were not associated with a higher risk of stroke/SE.
Robinson [17]	2021	Letter to the editor	1	Rivaroxaban	Oxcarbazepine Gabapentin Topiramate	Pulmonary embolism	PE in a patient with heterozygous prothrombin gene mutation, a history of PE, schizophrenia, psychosis, and bipolar disorder.
Sáez-Torres de Vicente [18]	2021	Letter to the editor	1	Rivaroxaban	Primidone	Stroke	Stroke in a patient with AF on treatment with primidone and rivaroxaban.
Candeloro [20]	2022	Retrospective cohort	32	Dabigatran Rivaroxaban Apixaban Edoxaban	Carbamazepine Phenytoin	Thrombotic event Major bleeding All-cause mortality	Patients with AF or VTE co-treated with a DOAC and carbamazepine or phenytoin had 4.4 per 100 person-year incidence of thromboembolism, 1.5 (95% CI, 1.2-1.9) per 100 person-year major bleeding, and 1.5 (95% CI, 1.2-1.9) per 100 person-year all-cause mortality.
Sulagna [13]	2022	Congress abstract	1	Apixaban	Phenytoin	Deep -vein thrombosis	60-y-old man with a history of DVT and PE on warfarin and phenytoin was switched to apixaban 5 mg twice daily. After 6 mo, he developed a new DVT episode.

AE, antiepileptic; AF, atrial fibrillation; DOAC, direct oral anticoagulant; DVT, deep vein thrombosis; JAK2, Janus kinase; OR, odds ratio; PE, pulmonary embolism; P-gp, P-glycoprotein; RR, rate ratio; SE, systemic embolism; VKA, vitamin K antagonist; VTE, venous thromboembolism.

We retrieved no records for the following AE drugs: ethosuximide, brivaracetam, clobazam, eslicarbazepine, felbamate, perampanel, tiagabine, vigabatrin, rufinamide, tiagabine, vigabatrin, and cenobamate.

The quality was found to be low or moderate across the cohort studies included in the review (Supplementary Table 2). The “selection of the nonexposed cohorts” was determined to be at a high risk of bias in 83% of the cohort studies included (Supplementary Table 2).

No information on patients’ race/ethnicity was reported in the studies included in the review. The results for each DOAC are summarized below.

### 3.1 Dabigatran

In a case series by Barbar et al. [10], among 14 patients taking a DOAC, 3 were on treatment with dabigatran and a concomitant AE drug. None experienced an adverse outcome (cerebrovascular event and/or venous thrombosis) during an observation period of 10 months (range, 1-27 months) [10]. However, neither the dabigatran dose nor the concomitant AE drug or the dose was specified. Moreover, although 9 of the 14 patients were taking a DOAC for VTE, the DOAC’s indication for the remainder was not specified [10]. In a case report from Paskaleva et al. [11], a 30-year-old man with a new deep vein thrombosis (DVT) in the context of heterozygous prothrombin gene mutation who was receiving valproic acid (1000 mg daily) was switched to dabigatran (150 mg twice daily) one month after the diagnosis of DVT after a lack of clinical improvement and low levels on rivaroxaban. There was a persistent lack of clinical improvement, and owing to lower-than-expected dabigatran levels, anticoagulant therapy was changed to a vitamin K antagonist that eventually led to clinical improvement [11]. In a congress abstract, Shah et al. [12] reported the case of a 25-year-old man on treatment with phenytoin for recurrent seizures. The patient developed cerebral venous thrombosis, and therapy with dabigatran was started. Neither the dabigatran nor the phenytoin dose was reported. After an unspecified time, the patient experienced worsening cerebral venous thrombosis [12]. In a retrospective cohort of patients with AF taking DOACs, Wang et al. [21] found that the concomitant use of dabigatran and levetiracetam, phenytoin, or valproic acid resulted in a higher incidence of major bleeding events than therapy with dabigatran alone. The reported rate ratios (RRs) were 2.77 (95% CI, 2.30-3.60), 2.70 (95% CI, 2.08-3.50), and 3.13 (95% CI, 2.59-3.78) for levetiracetam, phenytoin, and valproic acid, respectively. Neither the dose of dabigatran nor that of the AE drugs was reported [21]. In 2020, Zhou et al. [19] performed a pharmacoepidemiologic screening study assessing drugs with possible DDIs in DOAC-treated patients. The authors estimated the RR of being co-treated with interacting drugs in patients who experienced at least one thrombotic event. Among 1,394 patients who were receiving dabigatran, they found an RR of 2.04 (95% CI, 1.02-4.05) for topiramate co-prescription [19]. In a prospective cohort from Giustozzi et al. [23], 15 patients with AF were taking dabigatran of which 8, 5, and 2 patients were also taking levetiracetam, valproic acid, and carbamazepine, respectively. After a median follow-up of 17.5 months, one patient on dabigatran, 110 mg twice daily, and levetiracetam, 250 mg twice daily, had a fatal stroke [23]. Gronich et al. [6] collected information on DDIs in patients with AF or VTE receiving a newly prescribed DOAC from 2010 to 2020 in the Clalit database. The authors matched 1,116 cases of stroke or systemic embolism with 21,685 event-free controls. Among cases, 14 (6%) were taking dabigatran along with one P-gp/CYP3A4 inducer, whereas the same figure among controls was 99 (2.3%) for a resulting adjusted odds ratio (OR) of 2.59 (95% CI, 1.44-4.65) [6]. More recently, we reported data from a retrospective cohort of patients with AF or VTE taking both a DOAC or VKA together with carbamazepine or phenytoin. Among 6 patients treated with dabigatran, 4 and 2 patients were concomitantly on carbamazepine and phenytoin, respectively. After a median follow-up of 28 months, none of them experienced a thrombotic event [20].

### 3.2 Rivaroxaban

In the study by Perlman et al. [22], the authors found an increased reporting rate of thromboembolic and ischemic events in patients co-treated with rivaroxaban (no doses were specified); one of phenytoin, phenobarbital, or carbamazepine compared with patients co-treated with rivaroxaban; and one of valproic acid, lamotrigine, or levetiracetam (reporting OR, 1.79; 95% CI, 1.50-2.12). Several case reports and letters to the editor addressed the possible association of rivaroxaban and different AE drugs with thrombotic events. In the study by Barbar et al. [10], none of the 4 patients taking rivaroxaban and one AE drug experienced an adverse event during the observation time.Click or tap here to enter text. Langenbruch et al. [15] reported on a 30-year-old patient with epilepsy following neonatal meningoencephalitis on long-term treatment with valproic acid 2000 mg daily and lamotrigine 200 mg daily. The patient experienced multiple DVT episodes in the context of protein S-deficiency, hyperhomocysteinemia, and elevated lipoprotein(a) and started treatment with rivaroxaban 20 mg daily. Despite the DOAC treatment, he experienced a new DVT episode. Serum rivaroxaban levels were below the expected range; hence, the valproic acid was replaced with perampanel. At the follow-up, the rivaroxaban serum levels increased [15]. Paciullo et al. [14] reported on a 69-year-old man with AF and Janus kinase 2 positive thrombocythemia on treatment with rivaroxaban 20 mg daily and levetiracetam 1500 mg per day for focal seizures. The patient experienced recurrent transient ischemic attacks, and the authors reported low plasma levels of rivaroxaban. After levetiracetam withdrawal and replacement with lacosamide, the rivaroxaban levels increased [14]. Paskaleva et al. [11] described a case of a 30-year-old man on treatment with valproic acid 1000 mg per day who started rivaroxaban 15 mg twice daily for 3 weeks, followed by 20 mg daily for a DVT episode in the context of a heterozygous prothrombin gene mutation. After one month, there was no clinical improvement, and the rivaroxaban serum levels were lower than expected [11]. Wang et al. [21] found a higher rate of major bleeding events among patients with AF taking rivaroxaban and one concomitant AE drug compared with those taking rivaroxaban alone. The reported RRs were 2.14 (95% CI, 1.81-2.54) for levetiracetam, 2.2 (95% CI, 1.76-2.77) for phenytoin, and 2.48 (95% CI, 2.12-2.91) for valproic acid. Neither the dose of rivaroxaban nor that of the AE drug was reported [21]. In a letter to the editor, a 28-year-old man was taking rivaroxaban 20 mg daily for the treatment of pulmonary embolism in the context of a heterozygous prothrombin gene mutation. [12] The patient was on treatment with oxcarbazepine 600 mg twice daily, gabapentin, and topiramate with diagnoses of schizophrenia, psychosis, bipolar disorder, and depression. The patient developed a new pulmonary embolism with undetectable serum levels of rivaroxaban [17]. In another letter to the editor, a case of an 80-year-old woman with AF on treatment with rivaroxaban 20 mg and primidone who developed an ischemic stroke was reported [18]. In an observational study, the co-prescription of rivaroxaban and phenytoin was associated with at least one thrombotic event among 4924 patients (RR 2.39; 95% CI, 1.33-3.29) [19]. In Giustozzi et al. [23], among 25 patients with AF taking rivaroxaban, 8 patients were also taking levetiracetam, 5 were on valproic acid, and 2 were on carbamazepine. In this study, 4 patients experienced a stroke—one patient was receiving rivaroxaban 15 mg with phenobarbital 100 mg, the second rivaroxaban 15 mg with phenobarbital 100 mg and levetiracetam 250 mg twice daily, the third rivaroxaban 15 mg and levetiracetam 500 mg twice daily, and the last rivaroxaban 20 mg and carbamazepine 400 mg twice daily. Two of these strokes were fatal [23]. In the study by Gronich et al. [6], 13 (3.7%) patients with stroke or systemic embolism and 124 (1.8%) event-free matched controls were taking rivaroxaban along with one P-gp/CYP3A4 inducer for a resulting adjusted OR of 2.02 (95% CI, 1.12-3.62).

In our retrospective observational cohort, 12 patients, (4 on concomitant carbamazepine and 8 on concomitant phenytoin) were taking rivaroxaban. In total, 2 of them (one on rivaroxaban 15 mg twice daily and the other on rivaroxaban 20 mg once daily) experienced a DVT while they were taking phenytoin [20].

### 3.3 Apixaban

In a case report from the study by Di Gennaro et al. [16], an 88-year-old man on treatment with carbamazepine (dose not reported) for chronic essential trigeminal neuralgia was started on apixaban 5 mg twice daily for nonvalvular AF. After one month, the patient developed a transient ischemic attack (TIA) [16].

Perlman et al. [22] found a reporting OR of 1.88 (95% CI, 1.41-2.48) of thromboembolic or ischemic events for patients co-treated with apixaban and one of carbamazepine, phenytoin, or phenobarbital compared with patients co-treated with apixaban and one of valproic acid, lamotrigine, or levetiracetam.

In the case series by Barbar et al. [10], among 5 patients on apixaban and one AE drug, none experienced an adverse event during the follow-up. In the retrospective cohort from Wang et al. [21], the concomitant use of apixaban and levetiracetam, phenytoin, and valproic acid resulted in a higher rate of major bleeding events than in patients treated with apixaban alone. The corresponding RRs were 2.61 (95% CI, 1.99-3.42), 2.59 (95% CI, 1.66-4.05), and 2.96 (95% CI, 2.24-3.90), respectively. Neither the dose of apixaban nor that of the AE drugs was reported [21]. In the study by Gronich et al. [6], 12 (2.3%) patients with a stroke or systemic embolism and 121 (1.2%) event-free matched controls were taking apixaban and one P-gp/CYP3A4 inducer for a resulting adjusted OR of 1.99 (95% CI, 1.10-3.63). In the cohort from Giustozzi et al. [23], 42 patients were taking apixaban for stroke prevention in AF. Of these patients, 23 were also taking levetiracetam, 6 valproic acid, 3 phenobarbital, 3 carbamazepine, and 7 other anticonvulsant drugs. During the follow-up period, 3 strokes and one TIA occurred. Three patients were on apixaban 5 mg twice daily and were taking valproic acid 500 mg once daily (TIA), levetiracetam 250 mg twice daily (stroke), and levetiracetam 500 twice daily (stroke), respectively. The remainder had a stroke on apixaban 2.5 mg twice daily and carbamazepine 400 mg twice daily [23].

In our study, among 18 patients taking apixaban (of whom 10 patients were on carbamazepine and 8 were on phenytoin), one experienced a stroke (apixaban 5 mg twice daily + carbamazepine 600 mg once daily) and another had an intracranial bleed (apixaban 5 mg twice daily + phenytoin 750 mg once daily). The outcome of all-cause mortality was assessed in our study. There was only one death in a patient receiving apixaban 5 mg twice daily in combination with phenytoin, and the death was secondary to sepsis [20].

Finally, in a case report from Sulagna et al. [13], a 60-year-old man with a history of DVT and pulmonary embolism post-inferior vena cava filter on treatment with apixaban 5 mg twice daily and phenytoin (dose not reported) experienced a recurrent DVT.

### 3.4 Edoxaban

In the case report from the study by Di Gennaro et al. [16], after experiencing a TIA while taking apixaban in combination with carbamazepine, the patient was switched to edoxaban 60 mg once daily. After a follow-up period of 18 months after edoxaban initiation, no further thrombotic events were reported [16].

In the study by Barbar et al. [10], among 2 patients taking edoxaban and one AE, none experienced an adverse event during the observation time. In the prospective cohort from the study by Giustozzi et al. [23], among 9 patients with AF treated with edoxaban, 2, 3, 2, 1, and 1, were on concomitant treatment with levetiracetam, valproic acid, phenobarbital, carbamazepine, and another AE drug, respectively. None of them experienced an adverse outcome during the follow-up period [23].

In our retrospective cohort, we retrieved data on 2 patients on edoxaban and carbamazepine and one patient on edoxaban and phenytoin. During the observation period, none of them experienced an adverse outcome [20].

## 4 Discussion

In the rapidly evolving field of DOAC DDIs, this review updates the results from Taha et al. [8] and summarizes new data on clinical adverse outcomes regarding DOAC and AE DDIs, particularly for edoxaban, the latest DOAC introduced to the market. Despite the low quality of the studies reviewed, there appears to be a possible increased incidence of thrombotic events among patients who are co-administered DOACs and AE drugs. Based on limited evidence, edoxaban may be a safer DOAC in combination with AE drugs, potentially owing to its lesser metabolism through CYP3A; however, the available data are not sufficient to exclude a potential DDI [20,23].

Regarding major bleeding, Candeloro et al. [20] found a low rate of this outcome in patients taking DOACs in combination with carbamazepine and phenytoin, which was similar to patients taking warfarin in combination with these same AE drugs. Although they did not have a comparator group, Giustozzi et al. [23] reported a similarly low risk of major bleeding in patients taking DOACs and AE drugs. Wang et al. [21], however, showed an increased risk of major bleeding in patients taking DOACs and AE drugs. These results contrast with the results of Candeloro et al.’s [20] and Giustozzi et al.’s [23] studies and the expected effect of the co-administration of DOACs and AE drugs. The authors discussed how valproic acid could increase the risk of bleeding and how in the presence of renal failure, levetiracetam could increase the risk of bleeding if co-administered with an anticoagulant [21]. However, the proposed causative explanations are weak, and targeted studies are needed to confirm these results. With all-cause mortality only reported in one study with a single reported death, no conclusions can be drawn regarding the impact of DOAC AE DDIs on this outcome.

Regarding AE drugs traditionally considered safer to be co-administered with DOACs, including levetiracetam and valproic acid, the results of our study suggest that they too may in fact be associated with an increased risk of thrombotic events. Because levetiracetam is not believed to induce P-gp or CYP3A4, the mechanism of this potential DDI is unknown [24]. Valproic acid may increase the thrombotic risk by inducing CYP3A4. These data, however, are limited, and this question requires further study.

It is important to acknowledge a number of important limitations of our study; most importantly, the lower quality of evidence is at risk of bias informing our results. Although we have included additional prospective (*n* = 1) and retrospective observational studies (*n* = 5), similar to the study by Taha et al. [8], our results are also based in part on case reports (*n* = 3) and letters to the editor (*n* = 2). These data are subject to publication bias with cases associated with unfavorable outcomes more likely to be reported than cases with no apparent impact on these outcomes. Moreover, these data were limited by multiple confounders including concomitant thrombophilias, other thrombotic risk factors [[13], [14], [15],^17^,^18^], and incomplete information about DOAC or AE drug dose and adherence. Three of the retrospective cohorts we analyzed were based on large numbers of patients from national or claims-based databases [6,19,22]. However, the methodological designs did not allow for more granular analysis and did not exclude residual confounding. Furthermore, in the study by Gronich et al. [6] P-gp/CYP3A4 inducers other than AE drugs (rifampicin and Hyperici herba) were included in the analysis. Despite the long follow-up time, the low sample size underpowered the results of our cohort [20]. The prospective design of the study from Giustozzi et al. [23] produced stronger estimates but was based on relatively small sample size, used a nonrandomized design, and did not account for potential residual confounding. Additional limitations of our study include the restriction of our search to the Embase and MEDLINE databases, and we may therefore have missed other applicable articles. However, Embase and MEDLINE are the most comprehensive medical literature databases, and we believe it is unlikely that we have missed any articles of importance. We did not search the Cochrane Library because we excluded systemic reviews and meta-analyses. We also excluded patients affected by COVID-19. Although this reduced the effect of a confounding pro-thrombotic factor, the risk of thrombotic events in patients with COVID-19 receiving DOAC and AE drugs remains unexplored. Finally, race or ethnicity data were not explicitly reported in any of the studies included in our review; however, the cohort studies included patients from various countries including, Canada, the United States, Italy, Israel, and Taiwan, and case reports represented patients from additional countries. Although no conclusions can be made regarding any differential effect of clinical outcomes from DOAC and AE DDIs based on the basis of race or ethnicity characteristics, the broad representation of patients from various nations presumably of differing races or ethnicity increases the generalizability of our findings.

We recommend that future studies on DOAC and AE DDIs consider and address several important confounders. First, we suggest that future studies include populations with clear and comparable baseline thrombotic risk, plan a sufficiently long follow-up, and consider possible interruptions/resumptions of anticoagulant and anticonvulsant treatments as time-dependent variables. Moreover, many patients receive other drugs in addition to AEs that also interact with DOACs, limiting the ability to attribute any adverse clinical outcomes to the DOAC AE DDI specifically and must be accounted for [25]. Dexamethasone, for example, is commonly co-prescribed in patients with brain tumors receiving DOACs and AEs and could amplify the DOAC AE DDI because it also induces P-gp and CYP3A4 [1,26].

In conclusion, there are several potential DDIs between DOACs and AE drugs, but current evidence is limited and statistically underpowered to draw definitive conclusions regarding clinical significance. Well-designed clinical studies are of utmost importance.
