# Supplementary material for: Drug-drug interactions between direct oral anticoagulants and anticonvulsants and clinical outcomes: A systematic review
Source: Res Pract Thromb Haemost. 2023 Mar 28;7(3):100137. doi: 10.1016/j.rpth.2023.100137 (PMC10131112; doi:10.1016/j.rpth.2023.100137)
Supplement: Supplementary material [file mmc1.docx]

**Supplementary Table 1. Ovid Search Strategy**

| OVID Medline Epub Ahead of Print, In-Process & Other Non-Indexed Citations, Ovid MEDLINE(R) Daily and Ovid MEDLINE(R) 1946 to Present | |
| --- | --- |
| 1 anticonvulsant.mp. or exp Anticonvulsants/ | 157677 |
| 2 (phenobarbital or phenytoin or primidone or valproate or carbamazepine or ethosuximide or brivaracetam or clobazam or eslicarbazepine or felbamate or gabapentin or pregabalin or lacosamide or lamotrigine or levetiracetam or oxacarbazepine or perampanel or tiagabine or topiramate or vigabatrin or zonisamide or rufinamide or cenobamate).mp. | 87222 |
| 3 exp Factor Xa Inhibitors/ or doac.mp. or noac.mp. | 12552 |
| 4 (rivaroxaban or apixaban or edoxaban or dabigatran).mp. | 13000 |
| 5 1 or 2 | 177790 |
| 6 3 or 4 | 18777 |
| 7 5 and 6 | 77 |
| 8 limit 7 to dt=20190301-20221201 | 33 |
| Embase <1996 to 2022 December 19> | |
| 1 exp anticonvulsive agent/ | 359532 |
| 2 (phenobarbital or phenytoin or primidone or valproate or carbamazepine or ethosuximide or brivaracetam or clobazam or eslicarbazepine or felbamate or gabapentin or pregabalin or lacosamide or lamotrigine or levetiracetam or oxacarbazepine or perampanel or tiagabine or topiramate or vigabatrin or zonisamide or rufinamide or cenobamate).mp. | 178613 |
| 3 exp rivaroxaban/ or exp dabigatran/ or exp apixaban/ or doac.mp. or noac.mp. | 40536 |
| 4 (rivaroxaban or apixaban or edoxaban or dabigatran).mp. | 40359 |
| 5 1 or 2 | 364102 |
| 6 3 or 4 | 43785 |
| 7 5 and 6 | 1389 |
| 8 limit 7 to dd=20190301-20221201 | 77 |
| 9 limit 7 to rd=20190301-20221201 | 764 |
| 10 8 or 9 | 841 |

**Supplementary Table 2. Quality Assessment of Cohort Studies**

| Study | Selection | | | | Comparability | Outcome | | |
| --- | --- | --- | --- | --- | --- | --- | --- | --- |
|  | Representativeness of the exposed cohort | Selection of the non-exposed cohort | Ascertainment of exposure | Demonstration that outcome of interest was not present at start of study | Comparability of cohorts based on the design or analysis | Assessment of outcome | Was follow-up long enough for outcomes to occur | Adequacy of follow up of cohorts |
| Perlman 2019 | * | - | - | - | * | - | - | - |
| Wang 2020 | * | - | - | * | * | - | - | - |
| Zhou 2020 | * | - | - | - | - | - | * | - |
| Giustozzi 2021 | * | * | * | * | * | * | * | * |
| Gronich 2021 | * | - | * | - | - | * | * | * |
| Candeloro 2022 | * | - | * | * | - | * | * | * |

Footnote: * item present; - item not present.
